# New Advances in Iron Metabolism, Ferritin and Hepcidin Research

**DOI:** 10.3390/ijms232314700

**Published:** 2022-11-25

**Authors:** Paolo Arosio

**Affiliations:** Department of Molecular and Translational Medicine, University of Brescia, 25123 Brescia, Italy; paolo.arosio@unibs.it

The interest in the regulation of iron metabolism has increased in recent years with the clarification of the mechanism by which hepcidin regulates systemic iron homeostasis and the discovery of ferritinophagy, the major mechanism of ferritin degradation, which plays a major role in intracellular iron homeostasis and ferroptosis. In addition, increasing evidence indicates that iron deregulation occurs in a number of disorders, including cancer and neurodegeneration. We launched this Special Issue to verify if new findings on ferritin and hepcidin research may improve our understanding of how iron metabolism is controlled to avoid the dangerous consequences of deficiency and excess. We are pleased that the issue was successful and interesting manuscripts were submitted describing original research or conducting reviews.

Some papers focused on ferritin. Wang et al. [1] analyzed some properties of ferritin from the shrimp *Marsupenaeus japonicus.* They found that ferritin resists concentrations of Cd^2+^ and Hg^2+^ that cause the aggregation of human ferritins. This difference was attributed to the position of Cys residues that are buried and not accessible in the shrimp ferritin but exposed in the human ferritin. This interesting property of shrimp ferritin might be exploited to remove heavy metal ions from contaminated food systems. Smith et al. [2] analyzed the mechanisms of iron release from ferritin in a milieu made of fresh yeast lysate in place of buffer to mimic more closely the conditions of cytosol where ferritin is normally localized. They found that, in this milieu, the rate of iron release was much faster than the one in buffer under the same conditions, suggesting that certain cellular metabolites of >50 kDa present in yeast cell lysate facilitate the reductive release of iron from the ferritin core. The rate of iron mobilization was reduced by the removal of NADPH and increased by the addition of physiological concentrations of free flavins, such as FMN, FAD, and riboflavin Altogether, the results indicate that, in addition to ferritinophagy and proteolysis, there exists an auxiliary iron reductive mechanism that involves long-range electron transfer reactions facilitated by the ferritin shell. Moreira et al. [3] studied the host response to *Mycobacterium avium* in mice, with attention to iron metabolism and ferritin. They found that the infection causes an increase in H, but not in L ferritin in the macrophages. They also found that mice deficient in FTH1 in myeloid cells are more resistant to the infection, with lower bacterial loads and lower levels of proinflammatory cytokines than the controls. Interestingly, the H ferritin produced by the myeloid cells was found in circulation possibly having a role in iron redistribution. The deletion of H ferritin in myeloid cells induced the expression of ferroportin and increased iron accumulation in hepatocytes. These results highlight the importance of FTH1 expression in myeloid cells for iron redistribution during infection.

Other studies concentrated on genetic disorders affecting iron homeostasis. One focused on the human SLC11A2 gene that encodes the divalent metal-iron transporter 1 (DMT1) that mediates iron absorption and recycling. Ultra-rare mutations of the gene are associated with hypochromic microcytic anemia with iron overload (AHMIO1). Romero-Cordadellas et al. [4] studied two novel cases of this disorder. One involved a splicing variant that was present in homozygosity. The second carried the G75R mutation, which was shown to cause DMT1 accumulation in lysosomes. It is suggested that erythropoietin could be a therapeutic approach for AHMIO1 patients to improve anemia and possibly contribute to mobilizing hepatic iron. The diagnosis of iron refractory iron deficiency anemia (IRIDA) caused by mutations of the TMPRSS6 gene is challenging due to its genotypical and phenotypical heterogeneity and is difficult to distinguish from multi-causal iron deficiency anemia (IDA). Van de Staaij et al. [5] studied whether the transferrin saturation (TSAT)/hepcidin ratio could be useful in this diagnosis. The IRIDA patients (n. 20) had a significantly lower TSAT/hepcidin ratio compared to the IDA controls (n. 39) at an optimal cut-off point of 5.6%/nM. The TSAT/hepcidin ratio showed excellent performance in discriminating IRIDA from *TMPRSS6*-unrelated IDA, provided that recent iron therapy and moderate-to-severe inflammation was absent. Rosato et al. [6] studied dyserythropoietic anemia type II (CDA II), a disorder hallmarked by ineffective erythropoiesis, hemolysis, erythroblast morphological abnormalities, and hypo-glycosylation of some red blood cell membrane proteins and severe iron overload caused by biallelic variants of the SEC23B gene. This *gene* encodes the cytoplasmic coat protein complex II (COPII) important for the endoplasmic reticulum to Golgi trafficking and protein glycosylation. They silenced the gene in HuH7 hepatic cell line and found that this caused alteration in BMP/SMAD pathway effectors and a reduced sensibility to BMP6 stimulus. This was caused by the impairment in the glycosylation of proteins involved in the activation of the BMP/SMAD pathway with subsequent hepcidin suppression. The findings suggested that iron overload in CDA II is associated with both ineffective erythropoiesis and the alteration of hepatic hepcidin expression.

The role of iron in neurodegeneration is an important topic analyzed in some papers. Raha et al. [7] investigated the distribution and expression of key iron proteins in the brain tissues of patients with Alzheimer’s disease (AD), Parkinson’s disease (PD), Huntington’s disease (HD), and Down syndrome (DS) dementia. Ferritin was found in senile plaques in the AD and DS brains, as well as in the Lewy bodies in PD brain. Ferroportin was strikingly reduced in the AD brain compared to the age-matched controls. Its downregulation could alter cellular iron entry and exit pathways of the endothelium and cause iron mismanagement. Extensive blood vessel damage in the basal ganglia and deposition of ferritin heavy chain (FTH) and hepcidin were found in the caudate and putamen in PD and DS brains. Membrane damage and subsequent impairment of ferroportin and hepcidin cause oxidative stress that contributes to neurodegeneration seen in DS, AD, and PD subjects. Ward et al. [8] reviewed present knowledge on the role of iron in neuroinflammation and neurodegeneration. The disturbance of brain homeostasis will activate microglia to synthesize a variety of pro-inflammatory agents that may lead to inflammation and cell death. These agents will induce changes in the proteins responsible for regulating iron homeostasis, thus increasing iron deposition in cells in the brain. The generation of reactive oxygen and nitrogen species involved in the inflammatory process can affect iron metabolism via their interaction with iron-regulatory proteins (IRPs). Therapeutic approaches to minimize the toxicity of iron include N-acetyl cysteine, non-steroidal anti-inflammatory compounds, and iron chelation. Porras and Rouault [9] presented an overview of the pathological consequences of iron metabolism disruption in CNS, which has increasingly been implicated in various neurological disorders. This review examined the consequences of both iron accumulation and deficiency in various disease contexts, including neurodegenerative, neurodevelopmental, and neuropsychological disorders. The history of animal models of iron metabolism misregulation is also discussed followed by a comparison of three patients with a newly discovered neurodegenerative disorder caused by mutations in iron regulatory protein 2.

Tian et al. [10] studied the relationship between iron metabolism and ageing-related diseases, including neurodegenerative diseases. During ageing, the accumulation of nonheme iron destroys the stability of the intracellular environment. The destruction of iron homeostasis can induce cell damage by producing hydroxyl free radicals, leading to mitochondrial dysfunction, brain ageing, and even organismal ageing. In this review, the authors briefly summarized the role of the metabolic process of iron in the body, discussed recent developments of iron metabolism in ageing and age-related neurodegenerative diseases, and finally, explored some iron chelators as treatment strategies for those disorders. Understanding the roles of iron metabolism in ageing and neurodegenerative diseases will fill the knowledge gap in the field. This review could provide new insights into the research on iron metabolism and age-related neurodegenerative diseases.

Other papers studied the regulation of erythropoiesis. Berezovsky et al. [11] were interested in the mechanism by which Erythropoietin (EPO) downregulates hepcidin expression via the erythroferrone (ERFE) secreted by erythroblasts expressing transferrin receptor 2 (TFR2). They found that, after the administration of a single dose of EPO, splenic ERFE expression increased at 4 h while liver hepcidin mRNA decreased at 16 h. Additionally, splenic TFR2 and TFR1 proteins increased after EPO treatment. EPO treatments increased the amount of TFR1 protein in plasma exosomes but not that of TFR2. The results confirm the importance of ERFE in stress erythropoiesis and support the role of TFR2 in erythroid cell development. Correnti et al. [12] reviewed the tight link that exists between iron metabolism and erythropoiesis. The coordination of erythropoietic activity and iron homeostasis is regulated by the liver-derived hormone hepcidin, which controls iron homeostasis via its interaction with the iron exporter ferroportin. When erythropoiesis is enhanced, iron availability to the erythron is mainly ensured by inhibiting hepcidin expression, thereby increasing ferroportin-mediated iron export from both duodenal absorptive cells and reticuloendothelial cells that process old and/or damaged red blood cells. Erythroferrone, a factor produced and secreted by erythroid precursors in response to erythropoietin, has been identified and characterized as a suppressor of hepcidin synthesis to allow iron mobilization and facilitate erythropoiesis.

Szymonik et al. [13] analyzed the characteristics of stemness markers and their influence on the development and course of neoplastic disease. In fact, a serious problem of many cancers is their resistance to anticancer drugs, which may be related to the properties of cancer stem cells (CSCs), which inhibit self-maturation for maintaining their self-renewal capacity and pluripotency. They increase the expression of transcription factors such as OCT4, SOX2, KLF4, Nanog, and SALL4. The metabolism of cancer cells demands an increase in iron and iron chelators have antitumor activity and influence the expression of stemness-related markers, thus reducing chemoresistance and the risk of tumor cell progression. This prompts the further investigation of these agents as promising anticancer novel drugs. Available iron chelators were also described, and their effects on cancer cells and expression of stemness-related markers were analyzed

Altogether, this Special Issue provides new data on the role and importance of ferritin in regulating intracellular iron and of hepcidin in controlling systemic iron homeostasis. It also shows advancements in the involvement of iron in some pathological conditions, including genetic disorders, neurodegeneration, and cancer.

## References

[1] Wang Y., Zang J., Wang C., Zhang X., Zhao G. (2021). Structural Insights for the Stronger Ability of Shrimp Ferritin to Coordinate with Heavy Metal Ions as Compared to Human H-Chain Ferritin. Int. J. Mol. Sci..

[2] Smith G.L., Srivastava A.K., Reutovich A.A., Hunter N.J., Arosio P., Melman A., Bou-Abdallah F. (2022). Iron Mobilization from Ferritin in Yeast Cell Lysate and Physiological Implications. Int. J. Mol. Sci..

[3] Moreira A.C., Silva T., Mesquita G., Gomes A.C., Bento C.M., Neves J.V., Rodrigues D.F., Rodrigues P.N., Almeida A.A., Santambrogio P. (2022). H-Ferritin Produced by Myeloid Cells Is Released to the Circulation and Plays a Major Role in Liver Iron Distribution during Infection. Int. J. Mol. Sci..

[4] Romero-Cortadellas L., Hernández G., Ferrer-Cortès X., Zalba-Jadraque L., Fuster J.L., Bermúdez-Cortés M., Galera-Miñarro A.M., Pérez-Montero S., Tornador C., Sánchez M. (2022). New Cases of Hypochromic Microcytic Anemia Due to Mutations in the *SLC11A2* Gene and Functional Characterization of the G75R Mutation. Int. J. Mol. Sci..

[5] van der Staaij H., Donker A.E., Bakkeren D.L., Salemans J.M.J.I., Mignot-Evers L.A.A., Bongers M.Y., Dieleman J.P., Galesloot T.E., Laarakkers C.M., Klaver S.M. (2022). Transferrin Saturation/Hepcidin Ratio Discriminates *TMPRSS6*-Related Iron Refractory Iron Deficiency Anemia from Patients with Multi-Causal Iron Deficiency Anemia. Int. J. Mol. Sci..

[6] Rosato B.E., Marra R., D’Onofrio V., Del Giudice F., Della Monica S., Iolascon A., Andolfo I., Russo R. (2022). SEC23B Loss-of-Function Suppresses Hepcidin Expression by Impairing Glycosylation Pathway in Human Hepatic Cells. Int. J. Mol. Sci..

[7] Raha A.A., Biswas A., Henderson J., Chakraborty S., Holland A., Friedland R.P., Mukaetova-Ladinska E., Zaman S., Raha-Chowdhury R. (2022). Interplay of Ferritin Accumulation and Ferroportin Loss in Ageing Brain: Implication for Protein Aggregation in Down Syndrome Dementia, Alzheimer’s, and Parkinson’s Diseases. Int. J. Mol. Sci..

[8] Ward R.J., Dexter D.T., Crichton R.R. (2022). Iron, Neuroinflammation and Neurodegeneration. Int. J. Mol. Sci..

[9] Porras C.A., Rouault T.A. (2022). Iron Homeostasis in the CNS: An Overview of the Pathological Consequences of Iron Metabolism Disruption. Int. J. Mol. Sci..

[10] Tian Y., Tian Y., Yuan Z., Zeng Y., Wang S., Fan X., Yang D., Yang M. (2022). Iron Metabolism in Aging and Age-Related Diseases. Int. J. Mol. Sci..

[11] Berezovsky B., Báječný M., Frýdlová J., Gurieva I., Rogalsky D.W., Přikryl P., Pospíšil V., Nečas E., Vokurka M., Krijt J. (2021). Effect of Erythropoietin on the Expression of Murine Transferrin Receptor 2. Int. J. Mol. Sci..

[12] Correnti M., Gammella E., Cairo G., Recalcati S. (2022). Iron Mining for Erythropoiesis. Int. J. Mol. Sci..

[13] Szymonik J., Wala K., Górnicki T., Saczko J., Pencakowski B., Kulbacka J. (2022). The Impact of Iron Chelators on the Biology of Cancer Stem Cells. Int. J. Mol. Sci..

